# The Effects of Human Milk Oligosaccharides on Gut Microbiota, Metabolite Profiles and Host Mucosal Response in Patients with Irritable Bowel Syndrome

**DOI:** 10.3390/nu13113836

**Published:** 2021-10-27

**Authors:** Cristina Iribarren, Maria K. Magnusson, Louise K. Vigsnæs, Imran Aziz, Ingvild Dybdrodt Amundsen, Tanja Šuligoj, Nathalie Juge, Piyush Patel, Maria Sapnara, Lea Johnsen, Nikolaj Sørensen, Johanna Sundin, Hans Törnblom, Magnus Simrén, Lena Öhman

**Affiliations:** 1 Department of Microbiology and Immunology, Institute of Biomedicine, Sahlgrenska Academy, University of Gothenburg, 40530 Gothenburg, Sweden; cristina.iribarren.gomez@gu.se (C.I.); maria.magnusson@microbio.gu.se (M.K.M.); maria.sapnara@gu.se (M.S.); johanna_sundin@hotmail.com (J.S.); 2 Department of Molecular and Clinical Medicine, Institute of Medicine, Sahlgrenska Academy, University of Gothenburg, 40530 Gothenburg, Sweden; imran.aziz1@nhs.net (I.A.); piyush.patel@gu.se (P.P.); hans.tornblom@gu.se (H.T.); magnus.simren@medicine.gu.se (M.S.); 3 DSM, Kogle Alle 4, 2970 Hørsholm, Denmark; louise.vigsnaes@helgum.dk (L.K.V.); Ingvild.Amundsen@dsm.com (I.D.A.); 4 Department of Technology, Faculty of Health, University College Copenhagen, 1799 Copenhagen, Denmark; 5 Department of Infection, Immunity and Cardiovascular Disease, University of Sheffield, Sheffield S10 2JF, UK; 6 Quadram Institute Bioscience, Norwich Research Park, Norwich NR4 7UQ, UK; tanja.suligoj@quadram.ac.uk (T.Š.); nathalie.juge@quadram.ac.uk (N.J.); 7 MS-Omics, 2950 Copenhagen, Denmark; lgj@msomics.com; 8 Clinical Microbiomics, 2100 Copenhagen, Denmark; Nikolaj@clinical-microbiomics.com; 9 Center for Functional GI and Motility Disorders, University of North Carolina, Chapel Hill, NC 27599, USA

**Keywords:** 2′-O-fucosyllactose, antibacterial response, gut microenvironment, human milk oligosaccharides, irritable bowel syndrome, lacto-N-neotetraose, metabolomics, microbiota

## Abstract

Background: Human milk oligosaccharide supplementation safely modulates fecal bifidobacteria abundance and holds the potential to manage symptoms in irritable bowel syndrome (IBS). Here, we aimed to determine the role of a 4:1 mix of 2′-O-fucosyllactose and lacto-N-neotetraose (2′FL/LNnT) on the modulation of the gut microbiota composition and host mucosal response, as well as the link between the bifidobacteria abundance and metabolite modulation, in IBS patients. Methods: Biological samples were collected from IBS patients (*n* = 58) at baseline and week 4 post-supplementation with placebo, 5 g or 10 g doses of 2′FL/LNnT. The gut microbiota composition, metabolite profiles and expression of genes related to host mucosal response were determined. Results: Moderate changes in fecal, but not mucosal, microbial composition (β-diversity) was observed during the intervention with higher dissimilarity observed within individuals receiving 10g 2′FL/LNnT compared to placebo. Both fecal and mucosal *Bifidobacterium* spp. increased after 2′FL/LNnT intake, with increased proportions of *Bifidobacterium adolescentis* and *Bifidobacterium longum.* Moreover, the intervention modulated the fecal and plasma metabolite profiles, but not the urine metabolite profile or the host mucosal response. Changes in the metabolite profiles were associated to changes in bifidobacteria abundance. Conclusion: Supplementation with 2′FL/LNnT modulated the gut microbiota, fecal and plasma metabolite profiles, but not the host mucosal response in IBS. Furthermore, the bifidogenic effect was associated with metabolite modulation. Overall, these findings support the assertion that 2′FL/LNnT supplementation modulate the intestinal microenvironment of patients with IBS, potentially related to health.

## 1. Introduction

Irritable bowel syndrome (IBS) is a common functional gastrointestinal (GI) disorder driven by complex multifactorial mechanisms [1,2], typically characterized by recurrent abdominal pain associated with altered bowel movements. IBS can be divided into clinical subtypes based on the predominant bowel habits, i.e., diarrhea, constipation or mixed bowel habits [3]. Patients with IBS may present alterations in the gut microbiota composition [4,5] and function [6,7], or activation of the mucosal immune system [8,9], in some cases correlated to severity [4] or psychological symptoms [10]. Therefore, IBS may be regarded as a gut–brain axis disorder, with the intestinal microenvironment as an important player [11]. 

Host–microbiota interactions are central for maintaining gut health [12], and the gut microbiota participates in the fermentation of undigested dietary components [13,14] and modulation of neuro-immune interactions [15]. Growing evidence suggests that the gut metabolome reflects the host–microbiota activity and also influences intestinal epithelial cells and host immune responses [15,16], potentially regulating mucosal barrier functions. Therefore, interventions aiming to modulate and improve an altered host-microbiota crosstalk, and thereby potentially manage IBS symptoms, have emerged as an attractive treatment strategy [17]. 

Human milk oligosaccharides (HMOs) are complex glycans naturally present in high concentrations (5–25 g/L) in human breast milk [18]. Following ingestion, HMOs may be minimally digested in the GI tract [19], absorbed intact into the circulation or excreted in urine [20]. However, the majority of HMOs reach the large intestine undigested where they act as a nutrient source for the microbial community [18]. HMOs promote the growth of specific bacteria and have the potential to provide a health benefit [17]. Indeed, HMOs have been shown to modulate microbiota and metabolite composition [21,22], promote intestinal epithelial barrier protection [22,23] and immune responses [21,22,24] and thus, may provide resilience against infections [25]. 

2′O-Fucosyllactose (2′FL) is one of the most abundant fucosylated HMOs, with a mean concentration of 2.38 g/L in breast milk, whereas lacto-N-neotetraose (LNnT) is a non-fucosylated HMO found in lower quantities (~0.28 g/L in breast milk) [26]. These neutral HMOs have been extensively studied for their role in infant development [27]. However, less is known on their role in children [28] and adults [29]. Accordingly, the well-established beneficial properties of HMOs in infants have prompted interest in exploring their potential to improve gut health in adults. A recent in vitro study using a dynamic human gut simulator showed that fermentation of fecal samples, obtained from a healthy adult donor, supplemented with either 2′FL, LNnT or a 4:1 mix of 2′FL and LNnT (2′FL/LNnT) promoted the modulation of microbiota and metabolite profiles, and specifically increased abundance of bifidobacteria and short-chain fatty acids (SCFAs) [22]. Furthermore, the resulting HMO-fermented products regulated claudins and secretion of pro-inflammatory cytokines of CaCo2 cells and the gut-on-a-chip model system [22]. These in vitro findings suggest that HMOs have the capacity to regulate the intestinal microenvironment and thereby modulate barrier and immune functions of the gut in adults. Furthermore, supplementation with 2′FL or LNnT, or a combination of both substrates, has been shown to beneficially increase fecal bifidobacteria and shape microbiota composition in healthy adults [29]. 

Although HMOs have been suggested as promising supplements for the management of patients with microbiota–gut–brain axis disorders [30], studies of HMOs in patients with IBS are still scarce. A large-scale open-label trial conducted in patients with IBS recently demonstrated that 2′FL/LNnT supplementation improved GI symptoms and quality of life [31]. In addition, our group recently reported in a placebo-controlled proof-of-concept study that a mix of 2′FL/LNnT is well tolerated and beneficially impacts fecal bifidobacteria abundance in IBS patients after a 4-week treatment period [32]. However, more detailed studies exploring the effects of 2′FL/LNnT on the intestinal microenvironment in patients with IBS are lacking. Here, we analyzed biological samples collected before and after the 4-week daily supplementation with a 4:1 mix of 2′FL/LNnT [32] to test the hypothesis that HMOs could modulate gut microbiota and metabolite profiles as well as the host mucosal response in individuals with IBS. Furthermore, we determined the link between HMO-induced bifidogenic effect and metabolite modulation throughout the intervention.

## 2. Material and Methods

### 2.1. Study Cohort

The study cohort has been previously described in detail [32] Briefly, female and male (18–75 years) patients fulfilling the Rome IV criteria for IBS were recruited from the specialized outpatient clinic for functional gastrointestinal (GI) disorders at Sahlgrenska University Hospital (Gothenburg, Sweden) and local advertising between September 2016 and April 2018. All recruited patients presented moderate or severe IBS symptoms at entry (IBS Symptom Severity Scale (IBS-SSS) ≥ 175), and we accepted IBS patients from all subtypes based on the predominant bowel habits. All patients provided written informed consent before the initiation of the study. Exclusion criteria are described in Supplementary Material S1. 

### 2.2. Study Design 

The study was approved by the Regional Ethical Review Board in Gothenburg (Reg. No. 548-16), as well as being registered at www.ClinicalTrials.gov (NCT02875847) (accessed on 24 September 2018). The study was conducted between September 2016 and July 2018. Glycom A/S (now DSM, Hørsholm, Denmark) was the sponsor. A phase II, parallel, double-blind, randomized, placebo-controlled study was conducted in adult IBS patients (*n* = 61 at randomization) as previously described [32]. Briefly, after a 2-week screening period, patients were randomized and equally allocated to receive either placebo, 5 g or 10 g doses of a 4:1 mix of 2′-O-fucosyllactose (2′FL) and lacto-N-neotetraose (LNnT) (2′FL/LNnT) daily for 4 weeks. Patients were simultaneously stratified based on IBS subtypes (IBS with predominant constipation (IBS-C), or diarrhea (IBS-D), or mixed bowel habits (IBS-M)) within each intervention group. DSM provided 5 g and 10 g doses of 2′FL/LNnT as an active product. The 4:1 ratio of the 2′FL/LNnT mix selected aimed for an approximate reflection of the proportions of 2′O-fucosyllactose and lacto-N-neotetraose (4:1) within human breast milk based on previous reports [33,34]. The placebo control was 5 g of glucose (Dextropur, Dextro Energy GmbH and Co., Krefeld, Germany). Patients were recommended to maintain their regular diet and medication throughout the study.

Following screening, the intervention period comprised two visits, at baseline and week 4, and included collection of biological samples. Fecal samples were collected using sample collection and cooling kits at home, not earlier than 4 days prior to the visits (baseline and week 4) and kept at −20 °C in freezers until handling. Additionally, mucosal colonic biopsies, without bowel preparation, were taken during sigmoidoscopy and preserved in Allprotect Tissue Reagent (Qiagen, Hilden, Germany) for microbiota analysis and RNA later solution (Thermo Scientific, Waltham, MA, USA) for gene expression analysis. Whole blood was drawn from the antecubital vein by standard venipuncture techniques and then processed to extract the plasma (heparin tube). The original study protocol did not include urine sampling, but urine collection was approved after amendment of the study protocol. As a result, urine was collected only for patients included in the study after (19th September 2017). All samples were collected at Sahlgrenska University Hospital (Gothenburg, Sweden) and stored at −80 °C after each visit until their use. Additionally, at baseline, all patients completed validated questionnaires, the IBS symptom severity scale (IBS-SSS) [35] and the Hospital Anxiety and Depression Scale (HADS) [36], to assess the severity of IBS and psychological symptoms. Patients were re-assessed for these parameters at the end of the intervention as previously reported [32]. See Supplementary Material S1: *Clinical questionnaires* for more details. 

### 2.3. Gut Microbiota Analysis

Microbiota composition of fecal samples and mucosal colonic biopsies were analyzed by 16S rDNA sequencing at the Clinical Microbiomics A/S (Copenhagen, Denmark). Briefly, samples were mechanically disrupted using horizontal bead beating on a Vortex-Genie 2 at 2700 rpm for 5 min and total bacterial DNA was isolated using NucleoSpin^®^ 96 (Macherey-Nagel, GmbH and Co. KG, Düren, Germany). Then, a 16S rDNA Polymerase Chain Reaction (PCR) amplified the V3–V4 region using universal bacterial 16S rDNA primers [37] with Illumina adapters attached. A second PCR was run and included the Nextera Index Kit V2 (Illumina). The sequencing was carried out on the Illumina MiSeq sequencer using the MiSeq Reagent Kit V3 (Illumina). The analyses of the sequence data were performed using USEARCH (version 10.0) [38], mothur (version 1.38) [39], and internal scripts created by Clinical Microbiomics A/S. Reads with 97% sequence identity were clustered as Operational Taxonomic Units (OTUs). The detailed protocol is described in Supplementary Material S2.

### 2.4. Determination of Bifidobacterium *spp.* Levels

Assignment of the species level was attempted for Operational Taxonomic Units designated as *Bifidobacterium (B.)* spp. using the Nucleotide Basic Local Alignment Search Tool (BlastN) software. *B. adolescentis* NC_008618; *B. animalis* subsp. *lactis* NC_012815; *B. bifidum* NC_014638; *B. catenulatum* NZ_AP012325; *B. dentium* NZ_AP012326; *B. gallinarum* NZ_JGYX01000004; *B. longum* NC_004307; and *B. pseudocatenulatum* NZ_AP012330 were selected as reference genomes. 

### 2.5. Non-Targeted Metabolomic Analysis

Metabolite profiling was performed using fecal, plasma and urine samples by MS-Omics (Copenhagen, Denmark). Fecal water, filtered urine and aqueous plasma were obtained as described in Supplementary Material S2. The resulting samples were analyzed by liquid chromatography-mass spectrometry (LC-MS) method using a UPLC system (Vanquish, Thermo Fisher Scientific, Waltham, MA, USA) coupled with a high-resolution quadrupole-orbitrap mass spectrometer (Q Exactive™ HF Hybrid Quadrupole-Orbitrap, Thermo Fisher Scientific). LC method was based on an adapted version of a previously established protocol [39]. Appropriate quality control (QC) samples were included throughout the analysis and analyzed in tandem mass spectrometry mode to allow the identification of compounds. 

The raw data were processed on Compound Discovered software Version 3.0 (Thermo Scientific, Thermo Fisher Scientific, Waltham, MA, USA) as described in Supplementary Material S2. All compounds were processed and classified into different annotation levels of decreasing confident identification as detailed in Supplementary Material S2. For further statistical analysis, annotation levels 1, 2a and 2b were used and all values below the limit of detection (LOD) were adjusted to the correspondent lowest value assigned to each compound. Compounds identified in >50% of the samples below LOD, were excluded from the analysis, together with compounds annotated as potential pharmaceutical drugs. Furthermore, peaks corresponding to 2′FL and LNnT manually extracted from the raw data were included in the corresponding metabolite profiles.

### 2.6. Gene Expression Analysis 

Detection of 84 target genes related to the innate immune response against bacteria (antibacterial genes) and five genes related to gut barrier function in mucosal colonic biopsies was carried out using a quantitative PCR array (qPCR). Briefly, total RNA was isolated from biopsies using the commercial kit of NucleoSpin^®^ RNA Kit (Macherey-Nagel^TM^) according to the manufacturer’s protocol. From RNA, the cDNA was synthesized using RT^2^ First Strand Kit (Qiagen). 

The antibacterial genes were detected using RT^2^ Profiler™ PCR Array Human Antibacterial Response (Qiagen, Hilden, Germany) with RT^2^ SYBR Green qPCR Mastermix (Qiagen) in a QuantStudio 12K Real-Time PCR System (Applied Biosystems™, Life Technologies, Carlsbad, CA, USA). The list of all targeted genes can be found in Supplementary Material S2: Table S1. The selected gut barrier function-related genes were amplified in reactions containing QuantiTect SYBR Green PCR Master Mix (Qiagen, Hilden, Germany) and the corresponding primers (see Supplementary Material S2: Table S2). The qPCR was then performed using an Applied Biosystems^®^ 7500 Real-Time PCR System (Thermo Fischer Scientific, Waltham, MA, USA). All gene expression results were determined using 2^−∆Ct^ method and normalized to the corresponding housekeeping genes. Similarly, the fold change (week 4/baseline) was determined using the 2^−∆∆Ct^ method. Further information about both protocols can be found in Supplementary Material S2.

### 2.7. Statistical Analyses

Comparisons of demographic characteristics at baseline between the intervention groups were performed based on normality of distribution using IBM SPSS Statistics for Windows, Version 27.0 (Armonk, NY, USA; IBM Corp). Between-group comparisons were performed based on the normality of distribution. A chi-square test was used for categorical variables. ANOVA with Bonferroni’s correction was used for continuous variables.

Univariate analyses were applied to identify patterns in a single variable (e.g., α- and β-diversity) of the intervention groups. A Kruskal–Wallis test followed by Dunn’s correction was used for between-group comparisons while Mann–Witney U test assessed pairwise comparisons between baseline and week 4. All analyses were carried out using GraphPad Prism 7 (GraphPad Software, La Jolla, CA, USA) and used *p* < 0.05 as cutoff for statistical significance. Data were presented as median, median (interquartile range) or median (10–90th), unless differently indicated.

For the gut microbiota analysis, the α-diversity was estimated to describe the variation of microbiota composition within the samples at baseline and week 4. The number of OTUs, related to the richness of the samples; and Shannon index, that includes both richness and evenness (relative abundance) were performed by Clinical Microbiomics A/S. The β-diversity (gut microbiota composition between samples) was estimated by Bray-Curtis dissimilarity [40] in RStudio (R version 4.0.3, “Bunny-Wunnies Freak Out”) and used rarefied relative sequence abundance. The potential effect of the 2′FL/LNnT intervention on the metabolite and host mucosal response profiles were explored using unsupervised analysis (principal component analysis (PCA)) in RStudio [41]. For this purpose, a fold change (week 4/baseline) of each variable was calculated. Further, the distance between centroids (score averages of each group) was estimated (Supplementary Material S3: *Multivariate analysis*). Last, orthogonal partial least squares-discriminant analysis (OPLS-DA) was performed to identify between-group differences on antibacterial response modulation throughout the intervention using SIMCA^®^ Software (version 16.0.2, MKS Umetrics AB, Umeå, Sweden), similar to previously described [32]. 

## 3. Results

### 3.1. Demographics and Clinical Characteristics of the Study Cohort

In total, 58 IBS patients receiving either placebo, or 5 g or 10 g of 2′FL/LNnT daily for 4 weeks, as previously reported [32], were included in this exploratory study. The demographics and clinical characteristics of the patients at baseline are presented in Table 1. Briefly, the cohort consisted of IBS patients of all subtypes, taking either placebo (*n* = 19), 5 g 2′FL/LNnT (*n* = 20) or 10 g 2′FL/LNnT (*n* = 19) for 4 weeks (Table 1). The distribution of gender, age, body mass index, IBS severity and subtype, and psychological symptoms were similar in the study groups (Table 1). All patients complied with the intervention and followed the dietary advice [32]. Biological samples were obtained at baseline and week 4, although urine samples were only provided by a limited number of patients. A total of 116 fecal samples, 116 colonic mucosal biopsies, 115 plasma samples and 76 urine samples were collected from the patients who completed the study. Samples were equally distributed between intervention groups and visits.

### 3.2. Effect of 2′FL/LNnT on Fecal and Mucosal Microbiota Profiles

**Figure 1:**
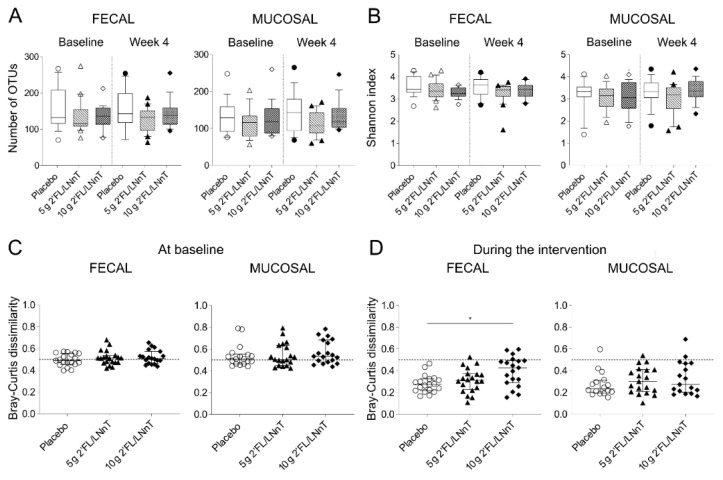
Microbiota diversity measures from fecal and mucosal samples throughout the intervention with 2′FL/LNnT or placebo in patients with irritable bowel syndrome (IBS). α-diversity, (**A**) Number of OTUs (operational taxonomical units) and (**B**) Shannon index at baseline and after 4-week intervention in placebo, 5 g 2′FL/LNnT and 10 g 2′FL/LNnT groups. β-diversity, (**C**) Between-patient microbiota dissimilarity at baseline and (**D**) within-patient microbiota dissimilarity in fecal samples and mucosal biopsies during intervention period in placebo, 5 g 2′FL/LNnT and 10 g 2′FL/LNnT group. 2′FL/LNnT, 4:1 mix of 2′-O-fucosyllactose and lacto-N-neotetraose. (**A**,**B**) Data are shown as median (10–90th percentile). (**C**,**D**) Dissimilarities were analyzed by Bray-Curtis dissimilarity index. Dashed line at y = 0.5 indicates intermediate dissimilarity. Data are shown as median (interquartile range). (**A**–**D**) Between-group comparisons calculated by Kruskal–Wallis test followed by Dunn’s correction for multiple comparisons. * *p* < 0.05.

We first investigated the effect of 2′FL/LNnT on gut microbiota richness and diversity. Species richness, determined as numbers of Operational Taxonomic Units (OTUs), of fecal and mucosal samples were comparable in all intervention groups at baseline and after the intervention (Figure 1A). Furthermore, Shannon index, a measurement of microbiota diversity based on numbers of OTUs and relative abundance, was similar in both sample types throughout the intervention (Figure 1B). Next, the fecal and mucosal β-diversity, describing the change in microbiota diversity between two communities, were assessed. Prior to the intervention, all groups presented with similar fecal and mucosal β-diversity (Figure 1C). Further, the fecal and mucosal β-diversity at baseline was not distinct from week 4 in any of the intervention groups (distance to group centroids and dispersion between groups, *p* > 0.05) (Supplementary Material S4: Figure S1). However, in terms of fecal microbiota, the change in microbiota composition within an individual overtime, was higher in the group who received 10 g 2′FL/LNnT compared with placebo (*p* < 0.05), whereas no differences between groups were detected with regards to mucosal microbiota dissimilarity (Figure 1D).

### 3.3. Effect of 2′FL/LNnT on Microbial and Bifidobacterial Communities 

**Figure 2:**
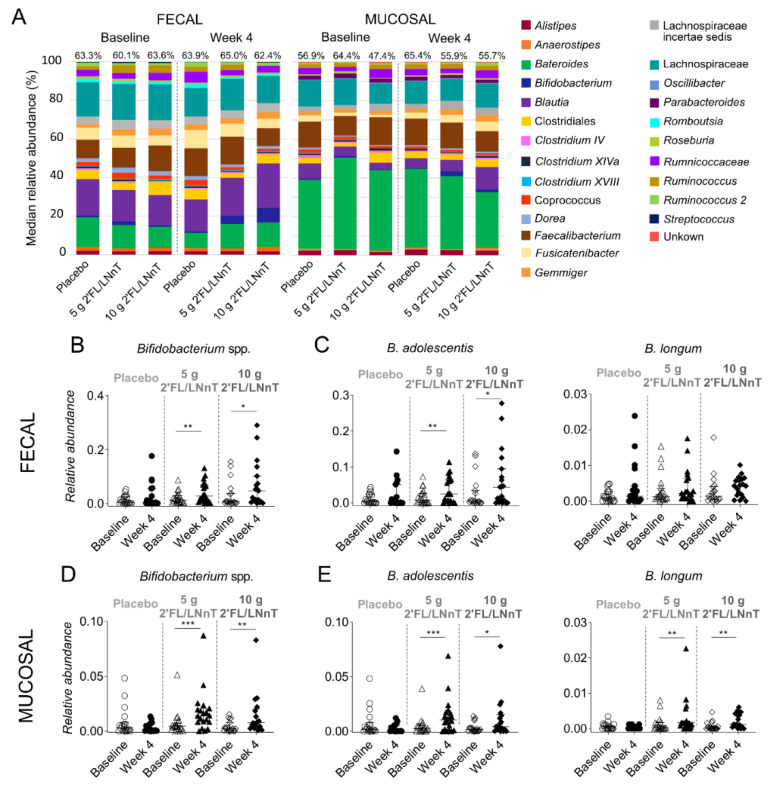
Microbiota profile and *Bifidobacterium* spp. throughout the intervention period with 2′FL/LNnT or placebo. (**A**) Relative abundance of the 23 most abundant OTUs detected in fecal and mucosal samples from placebo and 5 g 2′FL/LNnT and 10 g 2′FL/LNnT groups at baseline and week 4. Relative abundance of (**B**) fecal and (**D**) mucosal bifidobacteria at baseline and week 4 in placebo, 5 g 2′FL/LNnT and 10 g 2′FL/LNnT groups. (**C**) Relative abundance of fecal *B. adolescentis* and *B. longum* at baseline and week 4. (**E**) Relative abundance of mucosal *B. adolescentis* and *B. longum* at baseline and week 4. B., *Bifidobacterium.* (**A**) The median relative abundance is presented as percentage (%). OTUs are identified at genus level in *italics*, except the order Clostridiales, and the families Lachnospiraceae and Rumnicoccaceae. Percentages on top of each column indicate the proportion that these OTUs occupy in the whole microbiota composition detected. (**B**–**E**) Data shown as median (interquartile range), where each dot represents the relative abundance of each patient. Baseline (empty symbol) and week 4 (filled symbol) in placebo (circle), 5 g 2′FL/LNnT (triangle) and 10 g 2′FL/LNnT (diamond) group. Between-group comparisons calculated by the Kruskal–Wallis test followed by Dunn’s correction for multiple comparisons. * *p* < 0.05, ** *p* < 0.01, *** *p* < 0.001.

The most abundant OTUs were identified as each bacterial taxon that is present in more than 85% of the patient samples. The taxonomic analysis revealed that the 23 most abundant OTUs detected constituted 60.1–65.0% of the median abundance in fecal samples and 47.4–65.4% in mucosal biopsies. (Figure 2A). A high representation of the genera *Bacteroides, Blautia* and *Faecalibacterium* as well as the Lachnospiraceae family in both sample types were seen throughout the intervention (Figure 2A, Table 2 and Table 3). The relative abundance of some of these taxa changed after the intervention, as well as the relative abundance of other abundant OTUs detected in fecal samples and mucosal biopsies (Table 2 and Table 3). For instance, both active groups demonstrated a decrease of mucosal but not fecal *Bacteroides* at week 4 (*p* < 0.05). Moreover, fecal *Faecalibacterium* increased in 5 g 2′FL/LNnT group (*p* < 0.01) while it remained stable in the mucosa at week 4. Lastly, in the 10 g 2′FL/LNnT an increase of mucosal *Blautia* (*p* < 0.001) and a decrease of fecal Lachnospiraceae (*p* < 0.05) were recorded. Furthermore, supplementation with 5 g and 10 g 2′FL/LNnT, but not placebo, led to the increased relative abundance of fecal and mucosal bifidobacteria at week 4 as compared to baseline (*p* < 0.05 and *p* < 0.01) (Figure 2A,B,D, Table 2 and Table 3). Within *Bifidobacterium*, a total of five species were identified, and *Bifidobacterium (B.) adolescentis* and *B. longum* were present in more than 60% of the samples (Supplementary Material S4: Tables S3 and S4). Supplementation with 5 g and 10 g of 2′FL/LNnT increased the relative abundance of fecal *B. adolescentis* (*p* < 0.05), and mucosal *B. adolescentis* (*p* < 0.05) and *B. longum* (*p* < 0.01) at week 4 compared to baseline (*p* < 0.05) (Figure 2C,E). 

### 3.4. Effect of 2′FL/LNnT on the Metabolite Profile

**Figure 3:**
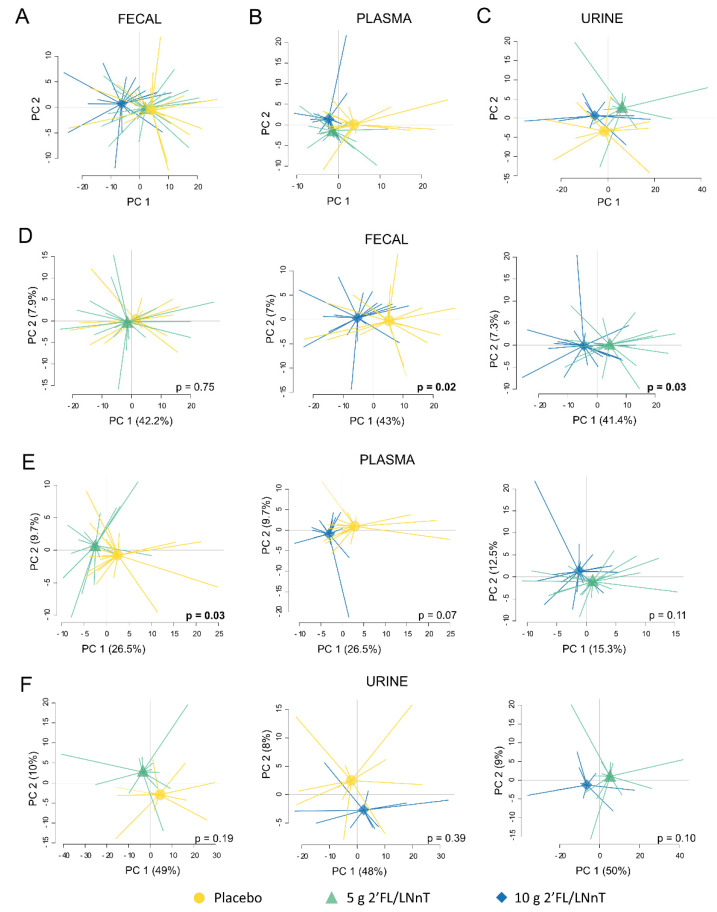
Metabolite profiles during the intervention period with 2′FL/LNnT or placebo. Principal component analysis (PCA) plots based on the fold-change (week 4/baseline) of (**A**) 384 fecal metabolites, (**B**) 217 plasma metabolites and (**C**) 528 urine metabolites in placebo (yellow circle), 5 g 2′FL/LNnT (teal triangle) and 10 g 2′FL/LNnT (blue diamond). Separate PCA plots presenting the profile fold-change of (**D**) fecal metabolites, (**E**) plasma metabolites, and (**F**) urine metabolites of the intervention groups in pairs: placebo and 5 g 2′FL/LNnT, placebo and 10 g 2′FL/LNnT and, 5 g and 10 g 2′FL/LNnT. The *p* values in (**D**–**F**) indicate statistical significance between the score group means (centroids).

Liquid chromatography-mass spectrometry allowed the detection of 384 metabolites in fecal samples, 217 plasma metabolites and 528 urine metabolites. At baseline, the three study groups did not differ with regards to fecal, plasma nor urine metabolite profiles (Supplementary Material S4: Figure S2). However, significant changes in the fecal metabolite profiles were detected, which were more marked in the intervention group which received 10 g 2′FL/LNnT as compared to the groups receiving 5 g 2′FL/LNnT or placebo (Figure 3A). Despite some overlaps, distinct fecal metabolite profiles of the intervention groups were confirmed in separate PCAs (Figure 3D). 3-Hydroxy-3-methylglutaric acid and N6-acetyl-L-lysine (compound nr 1733) were identified to have an important weight in the separation between the group receiving 10 g 2′FL/LNnT and the group receiving placebo, while N’-Hydroxy-2-(-1-naphthyl) ethanimidamide and N6-acetyl-L-lysine (compound nr 1733) were major contributors to the separation between both active groups. Furthermore, supplementation with 5 g of 2′FL/LNnT had the most pronounced effect on the plasma metabolite profile as compared to placebo (*p* < 0.05) (Figure 3B,E), while the 10 g dose showed a tendency in the same direction when compared to the placebo group (*p* = 0.07) (Figure 3E). Asparagine and 7-methulguanine were identified among the contributors to the separation between the groups receiving either 5 g 2′FL/LNnT and placebo. However, no differences in the urine metabolite profiles were seen between the intervention groups (Figure 3C,F). No peaks corresponding to 2′FL could be detected in the fecal samples at any time point whereas 2′FL was detected above the detection limit in a few plasma and urine samples at baseline. Furthermore, a significant increase was observed at week 4 in the patients that received 5 g and 10 g HMO, while the placebo group did not reveal such an increase (Table 4). Additionally, the peaks corresponding to LNnT were not detected in either fecal, plasma or urine samples in any of the intervention groups.

### 3.5. Link between Effect on Bifidobacterium spp. Abundance and Metabolite Modulation

**Figure 4:**
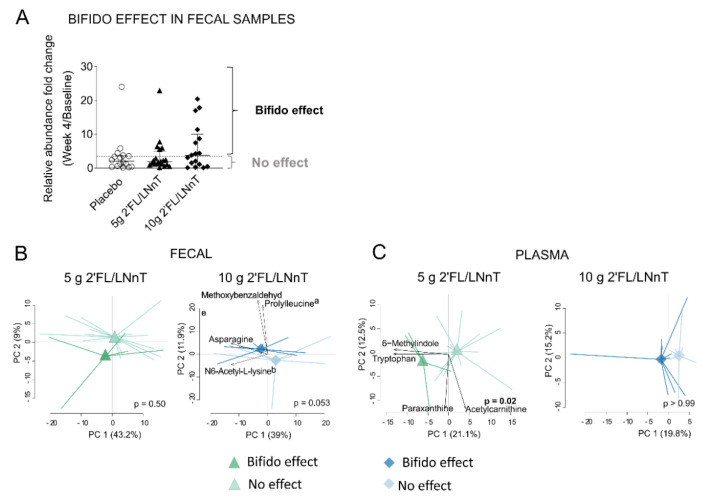
The metabolite profile modulation in relation to the effect of the 2′FL/LNnT on bifidobacteria (bifido effect). (**A**) Bifido effect based on the relative abundance fold change (week 4/baseline) of *Bifidobacterium* spp. in fecal samples. The bifido effect was defined as a fold change of bifidobacteria relative abundance during the intervention (week 4/baseline) greater than the upper quartile of the relative abundance fold change in the placebo group (week 4/baseline > 3.70). The lack of increase in bifido below the threshold was considered as “no effect”. (**B**) Principal component analysis (PCA) based on the fecal metabolite fold change in patients who presented a bifido effect (dark color) and no effect (light color) in the 5 g 2′FL/LNnT (left, teal triangles) and 10 g 2′FL/LNnT (right, blue diamonds). 5 g 2′FL/LNnT group: *n* = 5 bifido effect and *n* = 15 no effect; 10 g 2′FL/LNnT: *n* = 10 bifido effect; *n* = 8 no effect. ^a^ prolylleucine (compound nr 0163), ^b^ N6-Acetyl-L-lysine (compound nr 1733). (**C**) PCA based on the plasma metabolite fold change in patients who presented a bifido effect and with no effect in the 5 g 2′FL/LNnT and 10 g 2′FL/LNnT. 5 g 2′FL/LNnT group: *n* = 5 bifido effect and *n* = 15 no effect; 10 g 2′FL/LNnT: *n* = 10 bifido effect; *n* = 8 no effect. PCA plots with a clear separation between centroids show the loading of the most relevant metabolites involved in the observed pattern. The *p* values in (**B**,**C**) indicate statistical significance between the score group means (centroids).

The modulation of the metabolite profiles was further investigated with regards to the increased relative abundance of bifidobacteria in fecal samples. To this end, the patients were classified into two groups based on the fold change of relative abundance (week 4/baseline) of fecal bifidobacteria, using the upper quartile fold change of fecal bifidobacteria in the placebo group as cutoff (Figure 4A). Of the 54 classified patients (bifidobacteria relative abundance week 4/baseline > 0), 35% of IBS patients (placebo *n* = 4, 5 g 2′FL/LNnT group *n* = 5, 10 g 2′FL/LNnT group *n* = 10) had a relative abundance fold change of fecal bifidobacteria exceeding the mean fold change of the placebo group and were, therefore, considered as having an effect on bifidobacteria abundance (Figure 4A). Patients not meeting this criterion were considered to not have any bifidogenic effect following the intervention. The modulation of metabolite profiles was then compared between these patient groups. At baseline, the fecal metabolite profiles did not differ between the two groups of patients in any of the intervention groups (Supplementary Material S4: Figure S2). However, in the 10 g 2′FL/LNnT group, but not in the 5 g group, the fecal metabolite profile modulation tended to differ between patients displaying a bifidogenic effect (Figure 4B). Asparagine, prolylleucine (compound nr 0163), N6-acetyl-L-lysine (compound nr 1733), for example, were identified to have an important weight in the separation between the 10 g 2′FL/LNnT subgroups (Figure 4B). Furthermore, in the 5 g 2′FL/LNnT group, but not in the 10 g group, the plasma metabolite profile modulation differentiated depending on the bifidogenic effect, with for example tryptophan identified as an important factor for the separation (Figure 4C).

### 3.6. Effect of 2′FL/LNnT on the Host Mucosal Response Profile

**Figure 5:**
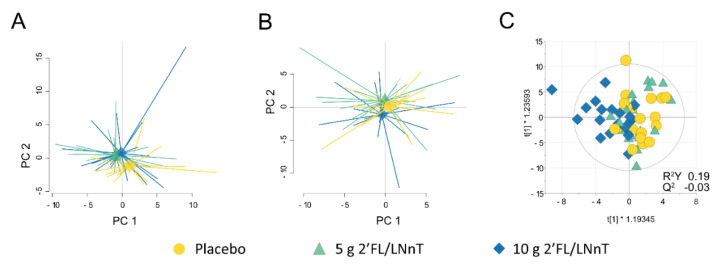
Antibacterial response gene expression profile during the intervention period with 2′FL/LNnT or placebo. Biopsies obtained from IBS patients before and after 4-week intervention were analyzed by polymerase chain reaction (PCR) array for 84 genes related to antibacterial response. (**A**) A principal component analysis (PCA) based on 80 unique genes (4 genes excluded due to expression below detection limit) at baseline in placebo (*n* = 19, yellow circle), 5 g 2′FL/LNnT (*n* = 20, teal triangle) and 10 g 2′FL/LNnT (*n* = 19, blue diamond) groups. (**B**) PCA and (**C**) orthogonal partial least squares-discriminant analysis (OPLS-DA) scatter plot of the antibacterial response gene expression profile log_2_ fold change (week 4/baseline) during the intervention between placebo, 5 g 2′FL/LNnT and 10 g 2′FL/LNnT groups. The R^2^ value determines goodness of the fit and the Q^2^ value represents the predictive ability of the model. In biological models, R^2^Y ≥ 0.5 and Q^2^ ≥ 0.4 are considered satisfactory.

The host mucosal response profile in colonic biopsies was determined, but no differences between the groups based on the antibacterial response gene expression profile (*n* = 80 expressed genes) before or throughout the intervention were identified (Figure 5A–C). The OPLS-DA did not provide a good model, as the R^2^Y < 0.5 and Q^2^ < 0 values indicated poor separation and predictability between the groups (Figure 5C). In addition, expression of genes related to tryptophan metabolism, mast cell activation and intestinal epithelial barrier function displayed comparable expression levels at baseline and after 4 weeks and was not modified due to intervention in any of the groups (Supplementary Material S4: Figure S3).

## 4. Discussion

This exploratory study of patients with IBS revealed that supplementation with 2′FL/LNnT changed the overall fecal, but not mucosal, microbiota profile within patients after a 4-week intervention period. The 2′FL/LNnT supplementation also shaped the relative abundance of specific bacterial taxa detected in fecal and mucosal samples, specifically fecal and mucosal *Bifidobacterium (B.) adolescentis* and mucosal *B. longum*. While 2′FL/LNnT supplementation modulated the fecal and plasma metabolite profiles, only 2′FL was detected in plasma and urine after the intervention, and a distinct metabolite modulation was linked to the effect of HMOs on bifidobacteria abundance throughout the intervention. In contrast to microbiota and metabolite profiles, the host mucosal response remained stable throughout the intervention period.

Little has been described about the effects of HMOs on gut microbiota composition in adults and most studies are based on infant-based studies or pre-clinical models. While fecal microbiota α-diversity was not altered in neonatal rats fed with 2′FL and controls [21], the fecal microbial α-diversity of infants fed with formula supplemented with 2′FL and LNnT became more similar to the microbiota profile of breastfed infants [42]. In our study, we did not see any effect of the 2′FL/LNnT supplementation on α-diversity or numbers of OTUs, as reflected by the unaltered Shannon index. However, 2′FL/LNnT supplementation modulated the fecal, but not mucosal, microbial β-diversity profile of patients in the group receiving the highest dose. The changes in the β-, but not in the α-diversity, might be related to the differences between these diversity metrics. The α-diversity evaluates the microbiota community described by the number of OTUs and relative abundance of individual samples [43]. Thus, the absence of α-diversity changes throughout the intervention suggests an equilibrium between the numbers of species present in the samples along with the intervention. In contrast, the β-diversity primarily captures changes in community composition [43] and a higher dissimilarity due to the intervention may reflect the niche competition and modulation of certain bacteria described below. The fact that 2′FL/LNnT supplementation influenced the fecal but not mucosal microbiota may be explained by the distinct microbiota profiles within the sampling locations [5], or by the fact that 2′FL/LNnT is mainly consumed by the luminal microbiota, potentially limiting the availability for mucosa-associated microbiota [44].

HMOs are directly consumed by certain *Bifidobacterium* spp. and *Bacteroides* spp. or utilized by other bacterial taxa by cross-feeding [45,46], inducing microbial modulation. In parallel, these changes may influence the abundance of other species due to niche competition [47]. In our study, we found changes in the relative abundance of the four most abundant OTUs *Faecalibacterium*, Lachnospiraceae and *Blautia* (Clostridiales order), previously described to be altered in IBS patients relative to healthy individuals [48,49]. Of these, an increase of *Faecalibacterium* after 2′FL/LNnT intervention, often found in low abundance in IBS, could be favorable in IBS management, whereas the benefit of detected changes of Lachnospiraceae and the genus *Blautia* to may be debated as there is still a lack of consensus in the so far few studies addressing the matter [48,49]. Several studies have also described increased abundance of *Bacteroides* (i.e., *Bacteroides fragilis*) in IBS compared to healthy controls (reviewed in [49]), a bacterial group considered to be efficient consumers of HMOs, at least in infants [45,46]. The ability of *Bacteroides fragilis* to degrade glycoproteins has been suggested to negatively influence the intestinal microenvironment, mucus production and intestinal motility, possibly triggering abdominal pain and diarrhea [49]. In our study, the relative abundance of the genus *Bacteroides* was reduced after 2′FL/LNnT intervention. Furthermore, *Bifidobacterium* spp., reported in lower levels in IBS patients [49], was increased following the 2′FL/LNnT supplementation, similar to previous reports in adults [29,32] and infants [42]. While the current study showed that both 5 g and 10 g doses induced growth of bifidobacteria, our previous work demonstrated similar effect only by the higher dose [32], a difference possibly explained by the different methods used for analyzing the microbiota (16S rDNA sequencing vs. GA-map analysis). The change of *Bifidobacterium*/*Bacteroides* ratio could be interpreted as being beneficial for IBS patients, and the mechanisms should be investigated in more detail in future studies. Nonetheless, the presence of an exogenous source of glycans that could indirectly prevent degradation of host mucosal glycoproteins, and the competition between 2′FL/LNnT consuming species [32] might be factors underlying such modulation. Additionally, various strains of bifidobacteria have different capacities to metabolize HMO [27,45,46]. For example, *B. longum* as well as *B. adolescentis* are known to utilize HMOs [27,50] and the latter has been identified as the main responder to 2′FL/LNnT supplementation [29]. Our intervention leading to an increased relative abundance of *B. adolescentis* in fecal samples and *B. longum* and *B. adolescentis* in mucosal biopsies supports these mechanistic data. Altogether, 2′FL/LNnT appears to contribute, directly or indirectly, to the modulation of specific bacterial taxa that may be involved in the pathophysiology of IBS and, therefore, of potential clinical benefit to this group of patients.

Metabolites reflect the function of the gut microbial community and influence host health [16]. Together with the microbiota sequencing, the metabolite profiling contribute to the understanding of the mechanisms of action of prebiotics [51]. For example, a dietary intervention based on a yogurt with symbiotic properties led to altered concentrations of serum metabolites such as acetone, choline, leucine and homocysteine, increased cell counts of fecal *Lactobacillus* and improved GI health in IBS-D patients [52]. The knowledge of the overall effects of HMO on the metabolite profile is currently scarce. Supplementation with 2′FL was demonstrated to change cecal microbiota and metabolite profiles and modulated gut-brain signaling in mice on a high-fat diet [53], and influenced cecal short-chain fatty acids and the urine metabolite profile in a rat model [21]. Moreover, fermentation of 2′FL, LNnT and 2′FL/LNnT shifted concentrations of short-chain fatty acids in a human sample based in vitro model system [22]. In our study, supplementation with 2′FL/LNnT modulated the metabolite profiles of fecal and plasma samples. While 5 g 2′FL/LNnT seemingly had a greater effect than 10 g dose on plasma samples, this was most likely due to the relatively small group sizes. The lack of effect in urine samples may be attributed to the fact that only half of the study subjects were included in this analysis, a limitation of this study. In addition, the distinct metabolite compositions of feces, plasma and urine, reflecting their diverse physiological functions, may explain that different 2′FL/LNnT doses have different impact on the metabolite profile modulation. Furthermore, the modulation of metabolite profiles differed between patients with and without an effect on bifidobacteria abundance. Interestingly, the distinct metabolite profile modulation was characterized by the change in fecal and plasma samples of asparagine, a metabolite thought to be important for maintaining intestinal barrier function [54], and tryptophan, an essential amino acid implicated in the pathogenesis of IBS [55]. One study showed that supplementation with *B. infantis* in rats increased tryptophan abundance in plasma, suggesting normalization of the tryptophan metabolism [55]. However, it needs to be acknowledged that the diet can also influence the metabolite profile and our study did not specifically control for dietary habits, except for advice to maintain the habitual diet throughout the study. The impact of the diet may complicate the interpretation of the metabolite modulation linked to a distinct effect on bifidobacteria abundance. Therefore, further investigation is needed to decipher the causality between the bifidogenic effect seen in this work following HMO supplementation and metabolite modulation.

Comparing breastfed infants with infants receiving formula demonstrated low but detectable levels of HMOs in plasma and urine [20], and under certain conditions also in feces [18]. In our study, 2′FL was detected in plasma and urine, but not in feces. The leakage of 2′FL into the circulation has been suggested to have systemic effects, and lead to lower plasma levels of inflammatory cytokines in breastfed infants and infants fed with formulas containing 2′FL [24]. Additionally, while the overall plasma and urine levels of 2′FL increased in both groups receiving 2′FL/LNnT, some patients had low amounts of 2′FL at week 4. The flexible time window, 0–3 days, between the last supplement intake and the collection of biological samples could explain the variation between patients within the same treatment group. Of note, identification of 2′FL in plasma and urine in patients supplemented with HMOs but not placebo, confirmed the study subjects’ adherence to the study protocol. In contrast, LNnT was absent in the three sample types. Despite being administered in a 4:1 ratio, the absence of LNnT in all samples, and the absence of 2′-FL in fecal samples, suggests that the compounds were utilized by gut microbiota such as for example *Bifidobacterium* spp. [18], which was found to increase in relative abundance during the intervention, when reaching the large intestine.

Previous reports support that in healthy individuals, HMOs regulate tight junction proteins [22], reduce permeability [22,23] and improve intestinal epithelial barrier function in vitro [22,23]. Furthermore, components of the host immune system such as pro-inflammatory cytokines [22,24] can be regulated by HMOs and associated with gut health. However, the effect of HMOs on the immune response in patients with IBS still needs to be explored. Our study did not identify changes in the host mucosal response, evaluated by the expression of genes related to antibacterial response and intestinal barrier integrity before and after 2′FL/LNnT intervention. A longer intervention period along with non-targeted RNA sequencing analysis may be required to detect effects on host mucosal response induced by 2′FL/LNnT supplementation.

This exploratory study provides proof-of-principle that 2′FL/LNnT supplementation imprints several layers of data and ecosystems, represented by the different sample types (i.e., feces, mucosal biopsies, plasma and urine). Compared to the GA-map analysis used in our previous study targeting 54 bacterial taxa related to GI disorders [32], the use of 16S rDNA sequencing provided new insights into the effect of HMOs in the gut microbiota composition of IBS patients. However, our study does not consider the potential interactions between the different players in the intestinal microenvironment, which might have provided an even better understanding of the effects of 2′FL/LNnT in host–metabolite–microbiota crosstalk in IBS. Therefore, a study integrating multiple layers of data, as previously suggested [17], will potentially offer an even more precise insight into the mechanisms behind 2′FL/LNnT supplementation. Additionally, this study did not allow us to relate the changes seen in microbiota or metabolite profiles to the improvement of the patients, since the original clinical trial evaluated safety and was not aimed at assessing the improvement of IBS symptoms. Nevertheless, the intestinal microenvironment modulation described in this study, together with the improvement of IBS symptoms previously reported in an open label trial with 2′FL/LNnT [31], indicate that 2′FL/LNnT promote gut health by targeting bifidobacteria, and calls for further investigations in a larger cohort.

In summary, we showed that a 4-week supplementation with 2′FL/LNnT shaped the gut microbiota in IBS patients, and specifically increased fecal and mucosal *B. adolescentis* and mucosal *B. longum*. Additionally, supplementation with 2′FL/LNnT modulated the fecal and plasma metabolite profiles, but did not influence host mucosal responses throughout the intervention period. Furthermore, a distinct metabolite modulation was linked to the bifidogenic effect throughout the intervention. Overall, our findings suggest that 2′FL/LNnT supplementation might be a valuable strategy to improve the intestinal microenvironment in IBS patients, although further studies are needed to decipher the underpinning mechanisms and evaluate possible beneficial effects on IBS symptoms.

## Figures and Tables

**Table 1:** Demographic data of the study cohort at baseline.

	Placebo (n = 19)	5 g 2′FL/LNnT (n = 20)	10 g 2′FL/LNnT (n = 19)	*p* Value
Sex (Female:Male)	14:5	11:9	14:5	0.35
Age, years †	45 (21−71)	42 (19−67)	47 (26−73)	0.65
Body mass index, kg/m^2^ †	24.7 (20.3−35.5)	24.0 (19.1−41.7)	24.4 (17.4−33.54)	0.79
IBS subtype ^¶^				
IBS-C	5	5	4	0.93
IBS-D	8	9	8	0.96
IBS-M	6	6	7	0.95
IBS-SSS ^¶^				
Mild	5	4	5	0.93
Moderate	7	11	5	0.30
Severe	7	5	9	0.57
HADS ^¶^				
Anxiety:No anxiety	8:15	8:12	6:12	0.59
Depression:No depression	1:18	3:17	3:15	0.86

2′FL/LNnT, 4:1 HMO mix of 2′-O-fucosyllactose and lacto-N-neotetraose. IBS-C; irritable bowel syndrome (IBS) with predominant constipation; IBS-D, IBS with predominant diarrhea; IBS-M, IBS with mixed bowel habits. IBS-SSS, Irritable Bowel Syndrome-Symptom Severity Scale; HADS, Hospital Anxiety and Depression Scale. † Mean and range (min–max). ^¶^ Number of patients. Differences between groups shown in bold (*p* < 0.05). IBS-SSS scores: mild (<174); moderate (175–300); severe (>300). HADS questionnaire: anxiety ≥ 8 (clinically relevant symptoms); no anxiety < 8; depression ≥ 8 (clinically relevant symptoms); no depression < 8.

**Table 2:** Relative abundance of the most abundant OTUs that were identified to be different in fecal samples between baseline and week 4 in the groups receiving either placebo, 5 g 2′FL/LNnT and 10 g 2′FL/LNnT.

	Placebo		5 g 2′FL/LNnT		10 g 2′FL/LNnT	
	Baseline (*n* = 19)	Week 4 (*n* = 19)	Baseline (*n* = 20)	Week 4 (*n* = 20)	Baseline (*n* = 19)	Week 4 (*n* = 19)
*Bifidobacterium*	5 × 10^−3^ (0–0.05)	5 × 10^−3^ (0–0.18)	0.01 (1 × 10^−3^–0.09)	**0.03** (2 × 10^−3^–0.13) **	7 × 10^−3^ (0–0.16)	**0.05** **(1 × 10^−3^–0.30)** *
*Clostridium*_XIVa	5 × 10^−3^ (6 × 10^−4^–0.06)	6 × 10^−3^ (4 × 10^−4^–0.05)	5 × 10^−3^ (6 × 10^−4^–0.19)	**3 × 10^−3^** (3 × 10^−4^–0.08) *	2 × 10^−3^ (0–0.03)	2 × 10^−3^ (8 × 10^−4^–0.02)
*Dorea*	0.01 (3 × 10^−3^–0.03)	0.01 (3 × 10^−3^–0.07)	0.02 (0–0.04)	9 × 10^−3^ (4 × 10^−3^–0.04)	0.01 (4 × 10^−3^–0.05)	**9 × 10^−3^** **(8 × 10^−4^−0.03)** *
*Faecalibacterium*	0.06 (0.03–0.21)	0.09 (0.02–0.19)	0.06 (2 × 10^−4^–0.12)	**0.09** (0–0.19) **	0.08 (0.08–0.20)	0.06 (8 × 10^−3^–0.19)
Lachnospiraceae	0.11 (0.04–0.24)	0.09 (0.03–0.31)	0.11 (0.02–0.25)	0.11 (0.02–0.21)	0.12 (0.02–0.42)	**0.09** **(0.02–0.17)** *
*Ruminococcus*	0.01 (0–0.05)	0.02 (0–0.06)	0.02 (0–0.09)	**0.02** **(2 × 10^−4^–0.08)** *	0.02 (0–0.08)	0.02 (2 × 10^−4^–0.06)

B, Baseline; W4, Week 4. Data shown as median (min–max). Significant differences in bold; * *p* < 0.05, ** *p* < 0.01.

**Table 3:** Relative abundance of the most abundant OTUs that were identified to be different in mucosal biopsies between baseline and week 4 in the groups receiving either placebo, 5 g 2′FL/LNnT and 10 g 2′FL/LNnT.

	Placebo		5 g 2′FL/LNnT		10 g 2′FL/LNnT	
	Baseline (*n* = 18)	Week 4 (*n* = 17)	Baseline (*n* = 19)	Week 4 (*n* = 20)	Baseline (*n* = 19)	Week 4 (*n* = 17)
*Anaerostipes*	5 × 10^−3^ (0–0.02)	6 × 10^−3^ (2 × 10^−4^–0.02)	3 × 10^−3^ (0–0.04)	3 × 10^−3^ (0–0.02)	3 × 10^−3^ (0–0.01)	**9 × 10^−3^** (4 × 10^−4^–0.01) **
*Bacteroides*	0.20 (0.04–0.47)	0.28 (0.05–0.39)	0.30 (2 × 10^−4^–0.53)	**0.21** **(0–0.55)** *	0.29 (0.03–0.45)	**0.16** **(7 × 10^−3^–0.39)** *
*Bifidobacterium*	3 × 10^−3^ (0–0.048)	1 × 10^−3^ (0–0.01)	5 × 10^−3^ (0–0.05)	**0.01** **(4 × 10^−4^–0.09)** **	2 × 10^−3^ (0–2 × 10^−3^)	**7 × 10^−3^** (6 × 10^−3^–0.08) **
*Blautia*	0.04 (0.01–0.09)	**0.04** **(9 × 10^−3^ −0.08)** *	0.03 (0.01–0.15)	0.03 (7 × 10^−3^ –0.2)	0.03 (8 × 10^−4^–0.07)	**0.06** **(2 × 10^−3^–0.17)** **
*Clostridium* XVIII	3 × 10^−3^ (0–8 × 10^−3^)	2 × 10^−3^ (0–6 × 10^−3^)	2 × 10^−3^ (0–9 × 10^−3^)	1 × 10^−3^ (0–3 × 10^−3^)	6 × 10^−4^ (0–7 × 10^−3^)	**3 × 10^−3^** **(0–0.01)** **
*Fusicatenibacter*	0.02 (6 × 10^−3^–0.08)	6 × 10^−3^ (0–0.05)	0.01 (2 × 10^−3^–0.04)	0.02 (2 × 10^−4^–0.11)	6 × 10^−3^ (2 × 10^−4^–0.07)	**0.03** (0–0.05) *
*Parabacteroides*	0.01 (0–0.06)	0.01 (0–0.09)	0.02 (0–0.09)	**9 × 10^−3^** **(0–0.1)** *	0.01 (0–0.21)	9 × 10^−3^ (0–0.07)
*Ruminococcus2*	5 × 10^−3^ (6 × 10^−4^ –0.03)	6 × 10^−3^ (0–0.02)	4 × 10^−3^ (0–0.02)	4 × 10^−3^ (0–0.02)	5 × 10^−3^ (0–0.04)	**9 × 10^−3^** **(0–0.04)** *

B, Baseline; W4, week 4. Data shown as median (min–max). Significant differences in bold * *p* < 0.05, ** *p* < 0.01.

**Table 4:** Levels of 2′FL in plasma and urine samples.

	Placebo		5 g 2′FL/LNnT		10 g 2′FL/LNnT	
	Baseline	Week 4	Baseline	Week 4	Baseline	Week 4
Plasma	7.0 (6.6–8.4)	6.8 (6.6–7.6)	6.8 (6.6–8.2)	**8.3** **(6.6–10.4)** ***	6.9 (6.6–8.0)	**8.9** **(6.2–10.9)** ***
Urine	7.0 (6.7–9.2)	7.1 (6.7–10.5)	6.9 (6.7–7.5)	**10.3** **(6.7–12.7)** **	6.85 (6.7–7.2)	**11.3** **(9.3–12.8)** **

Levels of 2′FL detected in plasma and urine samples at baseline and week 4 in each intervention group. Data expressed as natural logarithm of the peak area and shown as mean (min–max). The limit of detection of 2′FL was 6.6 peak area count in plasma samples and 6.7 peak area count in urine samples. Number of plasma samples: placebo group, *n* = 19 baseline and *n* = 19 week 4; 5 g 2′FL/LNnT group: *n* = 20 baseline and *n* = 20 week 4; 10 g 2′FL/LNnT group: *n* = 18 baseline and *n* = 19 week 4. Number of urine samples: placebo group, *n* = 11 baseline and *n* = 11 week 4; 5 g 2′FL/LNnT group: *n* = 12 baseline and *n* = 12 week 4; 10 g 2′FL/LNnT group: *n* = 11 baseline and *n* = 10 week 4. Significant differences in bold; ** *p* < 0.01; *** *p* < 0.001.

## Data Availability

The dataset used in this publication is available from the corresponding author on reasonable request.

## Supplementary Materials

The following are available online at https://www.mdpi.com/article/10.3390/nu13113836/s1, contains Supplementary Material S1: Study intervention. 1. Exclusion criteria and 2. Clinical questionnaires; Supplementary Material S2: Microbiome, metabolomic and host mucosal response analysis. 1. Gut microbiota analysis; 2. Non-targeted metabolomic analysis; 3. Gene expression analysis (Table S1 and Table S2); Supplementary Materials S3: Multivariate analysis; Supplementary Material S4: Figure S1. β-diversity measures from fecal and mucosal samples throughout intervention with 2′FL/LNnT or placebo in patients with irritable bowel syndrome (IBS); Table S3. Distribution of *Bifidobacterium* species in fecal samples at baseline and week 4; Table S4. Distribution of *Bifidobacterium* species in mucosal biopsies at baseline and week 4; Figure S2. Metabolite profiles prior the intervention with 2′FL/LNnT or placebo; Figure S3. Gene expression of gut barrier-related genes detected in mucosal biopsies by qPCR.
