# Supplementary material for: The Effects of Human Milk Oligosaccharides on Gut Microbiota, Metabolite Profiles and Host Mucosal Response in Patients with Irritable Bowel Syndrome
Source: Nutrients. 2021 Oct 27;13(11):3836. doi: 10.3390/nu13113836 (PMC8622683; doi:10.3390/nu13113836)
Supplement: Supplementary file 1 [file nutrients-13-03836-s001.zip › nutrients-1413532-supplementary.pdf]

SUPPLEMENTARY MATERIAL S1: STUDY INTERVENTION

1. EXCLUSION CRITERIA

The exclusion criteria included patients presenting with other GI diseases (e.g., lactose intolerance, inflammatory bowel disease) or other severe diseases. In addition, participation in another interventional study or intake of probiotic supplements and/or antibiotics up to a month before the screening visit or throughout the study were reasons for exclusion. Pregnant and nursing women were not included in the study.

2. CLINICAL QUESTIONNAIRES

Prior the intervention, the severity of IBS and psychological symptoms was assessed. For this purpose, patients completed IBS symptom severity scale (IBS-SSS) [1] and the Hospital Anxiety and Depression Scale (HADS) [2]. IBS-SSS measures abdominal pain intensity and frequency, severity of abdominal bloating/distension, dissatisfaction with bowel habits and life interference. The final score allows classification of patients according to symptom severity into mild (< 175), moderate (175 - 300) and severe (> 300) [1]. HADS questionnaire evaluates the severity of anxiety and depression with a score range from 0 to 21 on each of the two scales. HADS anxiety and depression scores ≥8 identify subjects with clinically relevant symptoms [2].

References

1. Francis, C.Y.; Morris, J.; Whorwell, P.J. The irritable bowel severity scoring system: a simple method of monitoring irritable bowel syndrome and its progress. *Aliment Pharmacol Ther* **1997**, *11*, 395-402. doi: https://doi.org/10.1046/j.1365-2036.1997.142318000.x.

2. Bjelland, I.; Dahl, A.A.; Haug, T.T.; Neckelmann, D. The validity of the Hospital Anxiety and Depression Scale. An updated literature review. *J Psychosom Res* **2002**, *52*, 69-77. doi: https://doi.org/10.1016/S0022-3999(01)00296-3.

SUPPLEMENTARY MATERIAL S2: MICROBIOME, METABOLOMIC AND HOST MUCOSAL RESPONSE ANALYSIS

1. GUT MICROBIOTA ANALYSIS

The 16S rDNA sequencing was carried out by Clinical Microbiomics A/S (Copenhagen, Denmark). Total bacterial DNA was isolated from fecal samples (0.1 g) and mucosal colonic biopsies (one biopsy specimen per patient) using the NucleoSpin 96 Stool and Tissue (Macherey-Nagel, GmbH & Co. KG) kits. The disruption of the samples by bead beating was done horizontally on a Vortex-Genie 2 at 2700 rpm for 5 min. At least one positive (ZymoBIOMICS™ Microbial Community Standard, Zymo Research) and one negative control were included with each batch of samples analyzed. Then, the V3-V4 region was amplified using 16S rDNA Polymerase Chain Reaction (PCR). The forward primer S-D-Bact-0341-b-S-17 and reverse primer S-D-Bact-0785- a-A-21 were used as universal bacterial 16S rDNA primers [3], with Illumina adapters attached. The PCR process included initial DNA denaturation at 98°C for 30 s, 25 cycles of denaturation and annealing as follows: 98°C for 10 s, 55°C for 20 s, 72°C for 20 s, and a final extension at 72 °C for 5 min. A similar PCR, but for 8 cycles, added index identifiers from the Nextera Index Kit V2 (Illumina). In both PCRs, amplification products and attachment of indices were verified on an agarose gel. Prior sequencing, amplification products from the nested PCR were pooled based on band intensity and cleaned with magnetic beads to form libraries; the DNA concentration was measured fluorometrically. Illumina MiSeq sequencer was used to carry out the sequencing using MiSeq Reagent Kit V3 (Illumina) for 2x 300 bp paired-end sequencing.

The analyses of the sequence data were performed using USEARCH (version 10.0) [4], mothur (version 1.38) [5], and internal scripts created by Clinical Microbiomics A/S. These analyses included tag identification, trimming and merging of paired-end reads with at least 20 bp overlap and a merged length of 400 - 500 bp. Low quality sequences, e.g., ambiguous bases, homopolymer length greater than 10, or more than one expected error were discarded and primer sequences trimmed. Clusters containing less than 5 identical sequences were also excluded. c*luster_otus* command used the most

abundant unique reads (dereplicated reads) as centroids to clustered sequences at 97 % sequence identity in USEARCH. Any suspected chimeras detected by internal comparison were discarded. OTUs were assigned to the correspondent taxonomic level using SINTAX with a cutoff value of 0.8 in opposition to the reference set "RDP training set" (version 16) [6].

2. NON-TARGETED METABOLOMIC ANALYSIS

Fecal, plasma and urine samples were processed and prepared for further analyses. Briefly, fecal water was prepared from fecal samples (100 g) mixed in two weight volumes of ultrapure water by high-speed centrifugation (16 000 g, at 4°C for 5 min). The resulting supernatant was transferred to a centrifugation filter for a second centrifugation (as above), and the filtrated supernatant was collected. Precipitation with methanol followed by liquid-liquid extraction with chloroform and water was performed in plasma samples. Then, the aqueous phase was collected and freeze-dried. Finally, the plasma samples were reconstituted in 200 µL 10mM ammonium formate with 0.1% formic acid. Last, filtered urine samples were obtained after 1:10 dilution of urine samples in 10mM ammonium formate with 0.1% formic acid, followed by filtration through a centrifugation filter. Appropriate quality control (QC) samples were prepared by pooling small volumes of each non-processed urine and blood samples or post- fecal sample extraction. QC-samples were analyzed regularly throughout the injection sequence.

Liquid chromatography–mass spectrometry (LC-MS) analysis was carried out using a UPLC system (Vanquish, Thermo Fisher Scientific) coupled with a high-resolution quadrupole-orbitrap mass spectrometer (Q Exactive™ HF Hybrid Quadrupole-Orbitrap, Thermo Fisher Scientific). An electrospray ionization interface was used in both negative and positive mode. QC-samples were analyzed in tandem mass spectrometry mode to allow identification of compounds. LC method, based on adapted version of a previously established protocol [39], was performed on the samples to analyze. The raw data was processed on Compound Discovered software Version 3.0 (Thermo Scientific, Thermo Fisher

Scientific, Waltham, MA, USA). Due to formation of different adducts, and appearance of different naturally occurring isomers a single compound is often represented by more than one feature with the same retention time but different masses. Consequently, for compound extraction, first peaks were detected and characterized by one mass and one retention time (identified as features). Next, features were grouped together if they belonged to the same compound. The accurate mass together with other additional information, e.g., isotope pattern, was used to determine the molecular formula. The information collected for each compound was used for later identification.

All compounds were processed for annotation and classified into different annotation levels of decreasing confident identification as follows. Level 1 was based on accurate mass, MS/MS spectra and known retention time from reference standards analyzed in the analytical sequence. Level 2 contained the sublevel 2a, based on accurate mass and known retention time obtained from a reference standard analyzed on the same system; and level 2b on accurate mass and MS/MS spectra from an external library. A third level based on library searches (i.e., human metabolome database) using accurate mass and elemental composition alone was performed, but together with unidentified compounds, were excluded from the post-analysis. The accurate mass was considered acceptable with a deviation of ± 0.0003 Da. For further statistical analysis, annotation levels 1, 2a and 2b were used and all values below the limit of detection (LOD) were adjusted to the correspondent lowest value assigned to each compound. Compounds identified in > 50% of the samples below LOD, were excluded from the analysis, together with compounds annotated as potential pharmaceutical drugs. Furthermore, peaks corresponding to 2’-O-fucosyllactose (2’FL) and lacto-N-neotetraose (LNnT) manually extracted from the raw data were included in the corresponding metabolite profiles.

3. GENE EXPRESSION ANALYSIS

Host mucosal response was evaluated targeting 84 antibacterial genes and 5 genes related to gut barrier function in colonic mucosal biopsies using quantitative PCR (qPCR). Total RNA was isolated

from colonic mucosal biopsies using the commercial kit of NucleoSpin® RNA Kit (Macherey-Nagel™, Düren, Germany) according to the manufacturer's protocol. Then, cDNA was synthesized from RNA using RT^2 First Strand Kit (Qiagen, Hilden, Germany).

For the analysis of antibacterial genes, the resulting cDNA was mixed in RT^2 SYBR Green qPCR Mastermix (Qiagen) and then dispensed into 384-well PCR Arrays plates, from the kit named RT^2 Profiler™ PCR Array Human Antibacterial Response (Qiagen, Hilden, Germany). The PCR array plates were read in a QuantStudio 12K Real-Time PCR System (Applied Biosystems™, Life Technologies, Carlsbad, CA, USA). The mean reported value (dRN threshold) of all plates was calculated to normalize Ct values among plates, except for one plate that was excluded due to very low dRN value. All samples passed the quality checks for PCR Array reproducibility, RT efficiency and genomic DNA contamination. The mean of the housekeeping genes ACTB, B2M, HPRT1 and RPLP0 was calculated for each sample and used to normalize the gene expression of the target genes (Table S1). For the analysis of gut barrier function-related genes, cDNA (2.5 ng or 12.5 ng) was amplified in 25 µL reaction containing 0.5 µM of each primer and 12.5 µL of 2× QuantiTect SYBR Green PCR Master Mix (Qiagen, Hilden, Germany). Following, qPCR was performed using an Applied Biosystems® 7500 Real-Time PCR System (Thermo Fischer Scientific, Waltham, MA, USA). Primers for Tryptophan Hydroxylase 1 (TPH-1) were purchased from Qiagen. Primers for Protease Activated Receptor 2 (PAR-2), Mucin-2 (MUC-2), Zonula Occludens-1 (ZO-1) and 40S ribosomal protein S13 (RPS13) were synthesized by Eurofins (Luxembourg) (Table S2). The PCR product specificity was confirmed by melt curve analysis. RPS13 was used as housekeeping genes to normalize the data. All gene expression results were determined using $2^{-\Delta Ct}$ method. Prior to this, undetermined gene expression was removed from the dataset and Ct > 36 was adjusted to Ct = 38. Prior to statistical analysis, $2^{-\Delta Ct}$ values of target genes > 7.00 (corresponding to $Ct_{target\ genes} < 19$) were excluded from the final database.

Table S1. Genes related to human antibacterial response targeted in RT^2 PCR arrays

Gene symbol	Full name
AKT1	V-akt murine thymoma vial oncogene homolog 1
APCS[ϕ]	Amyloid P component serum
BIRC3	Baculoviral IAP repeat containing 3
BPI	Bactericidal/Permeability-increasing protein
CAMP	Cathelicidin antimicrobial peptide
CARD6	Caspase recruitment domain family, member 6
CARD9	Caspase recruitment domain family, member 9
CASP1	Caspase 1, apoptosis-related cysteine peptidase
CASP8	Caspase 8, apoptosis-related cysteine peptidase
CCL3	Chemokine (C-C motif) ligand 3
CCL5	Chemokine (C-C motif) ligand 5
CD14	CD14 molecule
CHUK	Conserved helix-loop-helix ubiquitous kinase
CRP[ϕ]	C-reactive protein
CTSG	Cathepsin G
CXCL1*	Chemokine (C-X-C motif) ligand 1
CXCL2*	Chemokine (C-X-C motif) ligand 2
DMBT1	Deleted in malignant brain tumors 1
FADD	Fas (TNFRSF6)-associated via death domain
HSP90AA1	Heat shock protein 90kDa alpha (cytosolic), class A member 1
IFNA1[ϕ]	Interferon alpha 1
IFNB1	Interferon beta 1

Gene symbol	Full name
IKBKB	Inhibitor of kappa light polypeptide gene enhancer in B-cells, kinase beta
IL12A	Interleukin 12A
IL12B	Interleukin 12B
IL18	Interleukin 18
IL-1B	Interleukin 1 beta
IL-6	Interleukin 6
IL8	Interleukin 8
IRAK1	Interleukin-1 receptor-associated kinase 1
IRAK3	Interleukin-1 receptor-associated kinase 3
IRF5	Interferon regulatory factor 5
IRF7	Interferon regulatory factor 7
JUN	Jun proto-oncogene
LBP	Lipopolysaccharide binding protein
LCN2*	Lipocalin 2
LTF	Lactotransferrin
LY96	Lymphocyte antigen 96
LYZ	Lysozyme
MAP2K1	Mitogen-activated protein kinase kinase 1
MAP2K3	Mitogen-activated protein kinase kinase 3
MAP2K4	Mitogen-activated protein kinase kinase 4
MAP3K7	Mitogen-activated protein kinase kinase kinase 7
MAPK1	Mitogen-activated protein kinase 1
MAPK14	Mitogen-activated protein kinase 14
MAPK3	Mitogen-activated protein kinase 3

Gene symbol	Full name
MAPK8	Mitogen-activated protein kinase 8
MEFV	Mediterranean fever
MPO	Myeloperoxidase
MYD88	Myeloid differentiation primary response 88
NAIP	NLR family, apoptosis inhibitory protein
NFKB1	Nuclear factor of kappa light polypeptide gene enhancer in B-cells 1
NFKBIA	Nuclear factor of kappa light polypeptide gene enhancer in B-cells inhibitor, alpha
NLRC4	NLR family, CARD domain containing 4
NLRP1	NLR family, pyrin domain containing 1
NLRP3	NLR family, pyrin domain containing 3
NOD1	Nucleotide-binding oligomerization domain containing 1
NOD2	Nucleotide-binding oligomerization domain containing 2
PIK3CA	Phosphoinositide-3-kinase, catalytic, alpha polypeptide
PRTN3$^{\Phi}$	Proteinase 3
PSTPIP1	Proline-serine-threonine phosphatase interacting protein 1
PYCARD	PYD and CARD domain containing
RAC1	Ras-related C3 botulinum toxin substrate 1
RELA	V-rel reticuloendotheliosis viral oncogene homolog A (avian)
RIPK1	Receptor (TNFRSF)-interacting serine-threonine kinase 1
RIPK2	Receptor-interacting serine-threonine kinase 2
SLC11A1	Solute carrier family 11 (proton-coupled divalent metal ion transporters), member 1
SLPI	Secretory leukocyte peptidase inhibitor
SUGT1	SGT1, suppressor of G2 allele of SKP1 (S. cerevisiae)

**Gene symbol**	**Full name**
TICAM1	Toll-like receptor adaptor molecule 1
TICAM2	Toll-like receptor adaptor molecule 2
TIRAP	Toll-interleukin 1 receptor (TIR) domain containing adaptor protein
TLR1	Toll-like receptor 1
TLR2	Toll-like receptor 2
TLR4	Toll-like receptor 4
TLR5	Toll-like receptor 5
TLR6	Toll-like receptor 6
TLR9	Toll-like receptor 9
TNF	Tumor necrosis factor
TNFRSF1A	Tumor necrosis factor receptor superfamily, member 1A
TOLLIP	Toll interacting protein
TRAF6	TNF receptor-associated factor 6
XIAP	X-linked inhibitor of apoptosis
ZBP1	Z-DNA binding protein 1

Housekeeping genes

**Gene symbol**	**Full name**
ACTB	Actin beta
B2M	Beta-2-microglobulin
GAPDH[¶]	Glyceraldehyde-3-phosphate dehydrogenase
HPRT1	Hypoxanthine phosphoribosyltransferase 1
RPLP0	Ribosomal protein, large, P0

List of genes related to antibacterial profile and housekeeping genes that were targeted in RT² Profiler™ PCR Array Human Antibacterial Response (Cat. no. 330231 PAHS-148ZA, Qiagen).

ᶲ Gene excluded from the analysis due to ≥17% of samples had very low gene expression (CT=38)

Source: Gene Table PAHS- 148ZA from https://www.qiagen.com › genetable › pahs-148za. ¶Genes excluded for normalization of the data due to wider variation

Table S2. Genes related to gut barrier function targeted in qPCR

Gene symbol	Gene name	Primer assay	Primer sequence
TPH-1	Tryptophan Hydroxylase 1	Hs_TPH1_1_SG Q Primer Assay	QT00045346
Tryptase	Tryptase Alpha/Beta 1	F	ATCGTGCACCCACAGTTCTAC
		R	CTTCACCTGCTTCAGAGGAAAT
PAR-2	Protease activated receptor 2	F	CCCTGAACATCACGACCTG
		R	AGGCTGGGAACAGAAAGACC
ZO-1	Zonula occludens-1	F	AAGTCACACTGGTGAAATCC
		R	CTCTTGCTGCCAAACTATCT
MUC-2	Mucin 2	F	ACTGCACATTCTTCAGCTGC
		R	ATTCATGAGGACGGTCTTGG
RPS13	Ribosomal Protein S13	F	CGAAAGCATCTTGAGAGGAACA
		R	TCGAGCCAAACGGTGAATC

References

1. Francis, C.Y.; Morris, J.; Whorwell, P.J. The irritable bowel severity scoring system: a simple method of monitoring irritable bowel syndrome and its progress. *Aliment Pharmacol Ther* **1997**, *11*, 395-402.doi: https://doi.org/10.1046/j.1365-2036.1997.142318000.x.
2. Bjelland, I.; Dahl, A.A.; Haug, T.T.; Neckelmann, D. The validity of the Hospital Anxiety and Depression Scale. An updated literature review. *J Psychosom Res* **2002**, *52*, 69-77.doi: https://doi.org/10.1016/S0022-3999(01)00296-3.
3. Klindworth, A.; Pruesse, E.; Schweer, T.; Peplies, J.; Quast, C.; Horn, M.; Glöckner, F.O. Evaluation of general 16S ribosomal RNA gene PCR primers for classical and next-generation sequencing-based diversity studies. *Nucleic Acids Res* **2013**, *41*, e1.doi: https://doi.org/10.1093/nar/gks808.
4. Edgar, R.C. SINTAX: a simple non-Bayesian taxonomy classifier for 16S and ITS sequences. *bioRxiv preprint* **2016**, 074161.doi: https://doi.org/10.1101/074161.
5. Schloss, P.D.; Westcott, S.L.; Ryabin, T.; Hall, J.R.; Hartmann, M.; Hollister, E.B.; Lesniewski, R.A.; Oakley, B.B.; Parks, D.H.; Robinson, C.J., et al. Introducing mothur: open-source, platform-independent, community-supported software for describing and comparing microbial communities. *Appl Environ Microbiol* **2009**, *75*, 7537-7541.doi: https://doi.org/10.1128/aem.01541-09.
6. Cole, J.R.; Wang, Q.; Fish, J.A.; Chai, B.; McGarrell, D.M.; Sun, Y.; Brown, C.T.; Porras-Alfaro, A.; Kuske, C.R.; Tiedje, J.M. Ribosomal Database Project: data and tools for high throughput rRNA analysis. *Nucleic Acids Research* **2013**, *42*, D633-D642.doi: https://doi.org/10.1093/nar/gkt1244.
7. Oksaken, J.; Blanchet, F.G.; Friendly, M.; Kindt, R.; Legendre, P.; McGlinn, D.; Minchin, P.R.; O'Hara, R.B.; Simpson, G.L.; Solymos, P., et al. vegan: Community Ecology Package. 2020.
8. Josse, J.; Husson, F. missMDA: A Package for Handling Missing Values in Multivariate Data Analysis. *J Stat Softw* **2016**, *70*, 1-31.doi: https://doi.org/10.18637/jss.v070.i01.
9. R Core Team. R: A language and environment for statistical computing. R Foundation for Statistical Computing: Vienna, Austria. 2020.

SUPPLEMENTARY MATERIAL S3

MULTIVARIATE ANALYSIS

For the gut microbiota analysis, rarefied relative sequence abundance was used to investigate the gut microbiota composition between samples (β-diversity). Diversity between patients (samples at baseline), within patients (baseline vs week 4 for each patient), and between baseline and week 4 for each intervention group was determined by Bray-Curtis dissimilarity using the vegan package [7] in RStudio (R version 4.0.3, "Bunny-Wunnies Freak Out") [8]. Bray-Curtis dissimilarity provides an index between 0 and 1, where 0 indicates that all species are shared (high similarity) and 1 means that the microbiota composition is completely different (high dissimilarity) between samples. The betadisper function was used to summarize the β-diversity of the gut microbiota (compositional differences) between the intervention groups and time points on a Principal Coordinates Analysis (PCoA).

The potential effect of the 2'FL/LNnT intervention on the metabolite and host response profiles were explored using unsupervised analysis in RStudio [9]. Such effect was evaluated as fold change (week 4/baseline) and normalized to natural logarithm, unless otherwise stated. Principal Component Analysis (PCA) was performed using the prcomp and pca2d functions of the pca3d R package. When required, missing values were imputed using the missMDA package [8]. Furthermore, statistical significance between the multivariate group means (centroids) was calculated simulating a null-distribution of centroid differences by repetitive randomization of the group labels. The real value was ranked within the generated null-distribution. A greater distance between centroids than the actual value was considered significantly different if $p < 0.05$.

References

1. Oksaken, J.; Blanchet, F.G.; Friendly, M.; Kindt, R.; Legendre, P.; McGlinn, D.; Minchin, P.R.; O'Hara, R.B.; Simpson, G.L.; Solymos, P., et al. vegan: Community Ecology Package. 2020. https://CRAN.R-project.org/package=vegan (Accessed on 2nd November 2020)

2. Josse, J.; Husson, F. missMDA: A Package for Handling Missing Values in Multivariate Data Analysis. *J Stat Softw* **2016**, *70*, 1-31. doi: https://doi.org/10.18637/jss.v070.i01.
3. R Core Team. R: A language and environment for statistical computing. R Foundation for Statistical Computing: Vienna, Austria. 2020. https://www.r-project.org/ (Accessed on 1st October 2020)

SUPPLEMENTARY MATERIAL S4

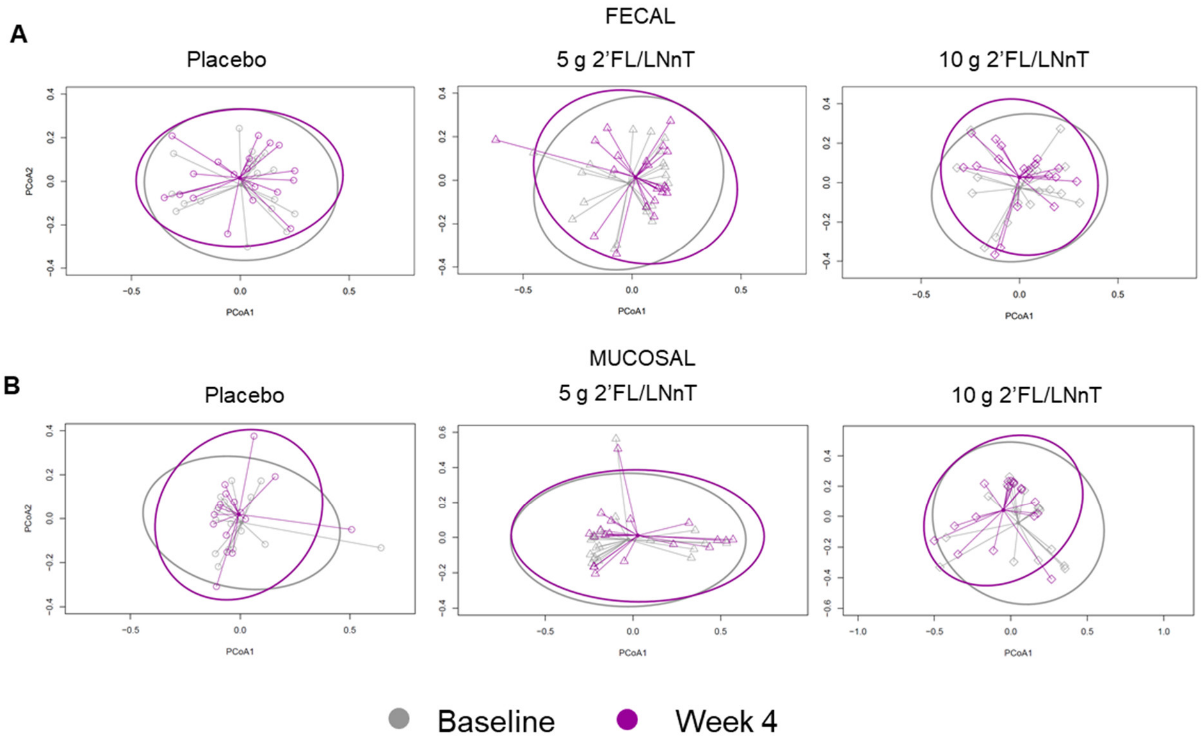

Figure S1. β-diversity measures from fecal and mucosal samples throughout intervention with 2'FL/LNnT or placebo in patients with irritable bowel syndrome (IBS). Principal Coordinate Analysis (PCoA) of Bray-Curtis distances showing dissimilarity of **(A)** fecal and **(B)** mucosal microbiota at baseline and week 4 in placebo, 5 g 2'FL/LNnT and 10 g 2'FL/LNnT group. Centroids represent the average score weighted, ellipses cover 95% confidence. Each dot is a subject at baseline (light grey) and week 4 (purple pink). 2'FL/LNnT, 4:1 mix of 2'-O-fucosyllactose and lacto-N-neotetraose.

	Placebo		5 g 2’FL/LNnT		10 g 2’FL/LNnT		
	Baseline (n = 19)	*Week 4* (n = 19)	*Baseline* (n = 20)	*Week 4* (n = 20)	*Baseline* (n = 19)	*Week 4* (n = 19)	*Total* (n = 116)
Bifidobacterium spp.	16 (84%)	16 (84%)	20 (100%)	20 (100%)	18 (95%)	19 (100%)	109 (93.9%)
Species level							
B. adolescentis	14 (73.7%)	15 (78.9%)	17 (85%)	18 (90%)	17 (89.5%)	16 (84.2%)	97 (83.6%)
B. animalis	0 (0%)	0 (0%)	2 (10%)	4 (20%)	0 (0%)	2 (10.5%)	8 (6.9%)
B. bifidum	3 (15.8%)	3 (15.8%)	6 (30%)	6 (30%)	4 (21.1%)	3 (15.8%)	25 (21.6%)
B. longum	13 (68.4%)	14 (73.7%)	19 (95%)	18 (90%)	17 (89.5%)	18 (94.7%)	99 (85.3%)
B. (pseudo)catenulatum$^{\varphi}$	5 (26.3%)	6 (31.6%)	3 (15%)	7 (35%)	8 (42.1%)	7 (36.8%)	36 (31%)
Unknown Bifidobacterium	3 (15.8%)	3 (15.8%)	1 (5%)	1 (5%)	0 (0%)	1 (5.3%)	9 (7.8%)

Table S3. Distribution of *Bifidobacterium* species in fecal samples at baseline and week 4.

No. of positive samples (% of total of samples). $^{\varphi}$*B. catenulatum* can also be identified as *B. pseudocatenulatum.* B., *Bifidobacterium;* 2’FL/LNnT, 4:1 mix of 2’-O-fucosyllactose and lacto-N-neotetraose.

	Placebo		5 g 2'FL/LNnT		10 g 2'FL/LNnT		
	Baseline (n=18)	*Week 4* (n=17)	*Baseline* (n=19)	*Week 4* (n=20)	*Baseline* (n=19)	*Week 4* (n=17)	*Total* (n=110)
Bifidobacterium *spp.*	13 (72.2%)	14 (82.4%)	17 (89.5%)	19 (95%)	14 (73.7%)	17 (100%)	94 (85.5%)
Species level							
B. adolescentis	12 (66.7%)	11 (64.7%)	14 (73.7%)	17 (85%)	11 (57.9%)	14 (82.4%)	79 (71.8%)
B. animalis	0 (0%)	1 (5.9%)	0 (0%)	1 (5%)	0 (0%)	0 (0%)	2 (1.8%)
B. bifidum	2 (11.1%)	4 (23.5%)	5 (26.3%)	6 (30%)	1 (5.3%)	1 (5.9%)	19 (17.3%)
B. longum	8 (44.4%)	8 (47.1%)	13 (68.4%)	16 (80%)	9 (47.4%)	14 (82.4%)	68 (61.8%)
B. (pseudo)catenulatum$^{\varphi}$	4 (22.2%)	3 (17.6%)	3 (15.8%)	6 (20%)	2 (10.5%)	5 (29.4%)	23 (20.9%)
Unknown Bifidobacterium	0 (0%)	0 (0%)	1 (5.3%)	1 (5%)	0 (0%)	1 (5.9%)	3 (2.7%)

Table S4. Distribution of *Bifidobacterium* species in mucosal biopsies at baseline and week 4.

No. of positive samples (% of total of samples). $^{\varphi}$*B. catenulatum* can also be identified as *B. pseudocatenulatum.* B., *Bifidobacterium;* 2'FL/LNnT, 4:1 mix of 2'-O-fucosyllactose and lacto-N-neotetraose.

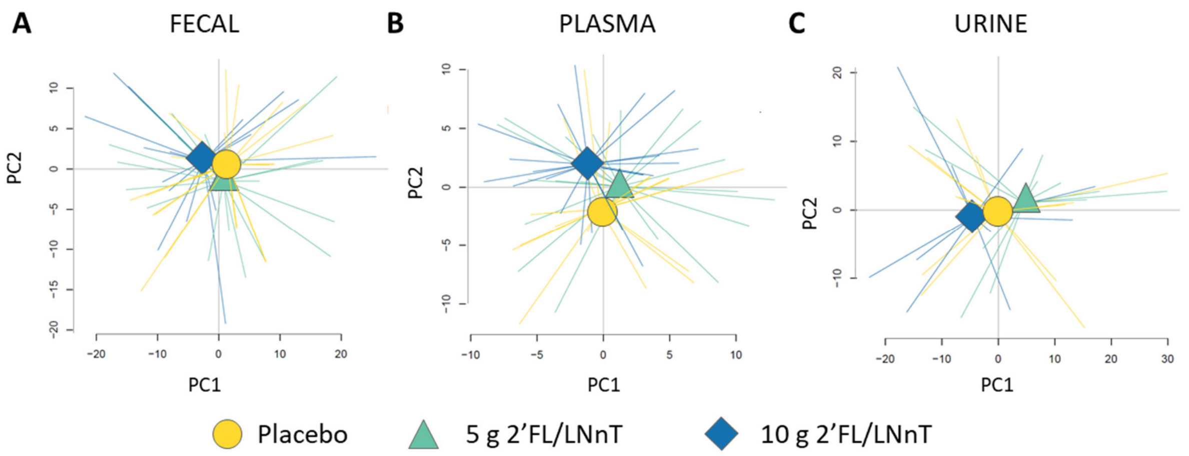

Figure S2. Metabolite profiles prior the intervention with 2’FL/LNnT or placebo. Principal Component Analysis (PCA) plots based on the profile of **(A)** 384 fecal metabolites, **(B)** 217 plasma metabolites and **(C)** 528 urine metabolites in placebo (yellow circle), 5 g 2’FL/LNnT (teal triangle) and 10 g 2’FL/LNnT (blue diamond) at baseline. 2’FL/LNnT, 4:1 mix of 2’-O-fucosyllactose and lacto-N-neotetraose.

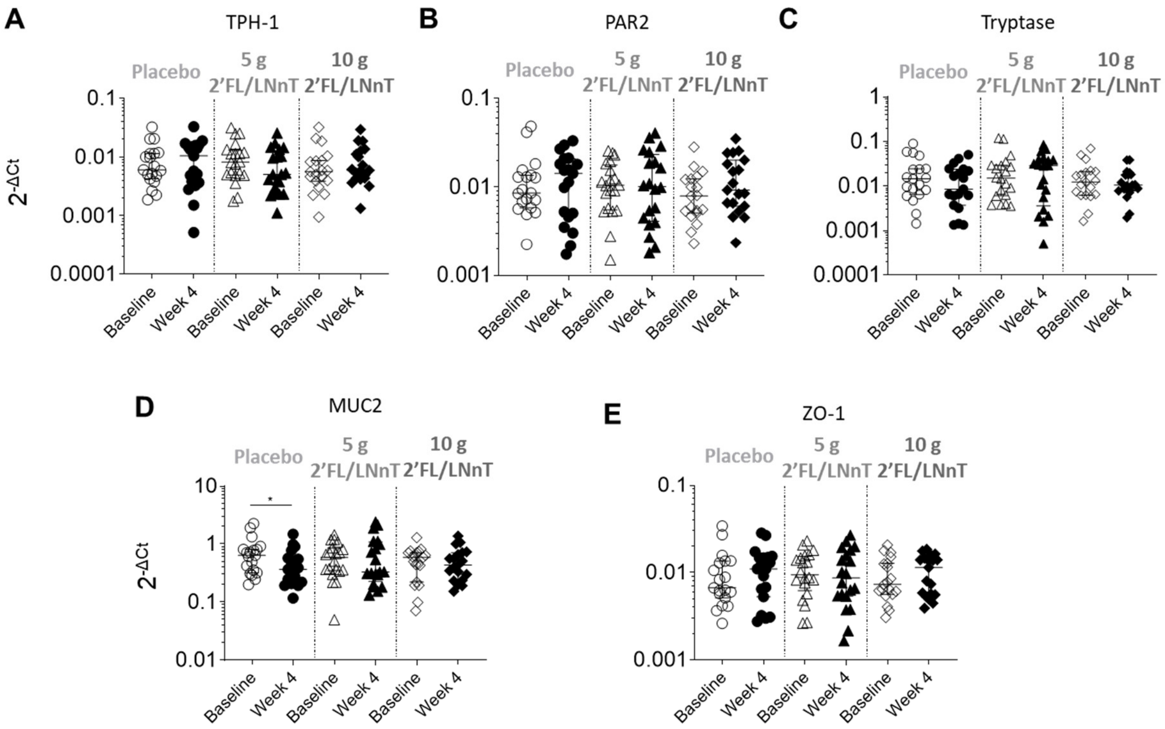

Figure S3. Gene expression of gut barrier-related genes detected in mucosal biopsies by qPCR. **(A)** tryptophan metabolism, **(B, C)** mast cell activation and **(D, E)** intestinal barrier function at baseline and week 4 in placebo, 5 g 2’FL/LNnT and 10 g 2’FL/LNnT groups. 2’FL/LNnT, 4:1 mix of 2’-O-fucosyllactose and lacto-N-neotetraose; TPH, Tryptophan Hydroxylase 1; PAR2, Protease Activated Receptor 2; MUC2, Mucin-2; ZO-1, Zonula Occludens-1. Data shown as median (interquartile range), $\log_{10}$ transformed. $^*p < 0.05$.
